# Accurate approximation to stochastic reaction diffusion on unstructured meshes in STEPS

**DOI:** 10.1186/1471-2202-16-S1-P55

**Published:** 2015-12-18

**Authors:** Iain Hepburn, Weiliang Chen, Erik De Schutter

**Affiliations:** 1 Theoretical Neurobiology, University of Antwerp, 2610 Antwerpen, Belgium; 2 Computational Neuroscience Unit, Okinawa Institute of Science and Technology Graduate University, Okinawa 904-0495, Japan

## 

Parallelization is vital in the future of spatial stochastic simulation and an important component of the Human Brain Project [1]. There are some unique challenges to be faced for simulators based on unstructured meshes such as STEPS [2] and finding the most accurate yet scalable solution for different biological systems is an ongoing area of exploration.

Scalable parallel solutions are known to require an approximation to diffusion that involves global execution of diffusion events at predetermined times, an approach that is termed "Operator Splitting" and was first applied for regular grids such as by the "Gillespie-Multi-Particle method" (GMP) [3]. Even serial implementations of GMP show a performance gain compared to exact solutions, and ~2 orders of magnitude gain has been demonstrated in a GPU implementation in a simple test system [4]. Therefore, the Operator Splitting method provides the best known parallel solution. However, it must be able to perform accurately in comparison to the exact spatial SSA for a wide range of simulation conditions, and this has not been extensively investigated before.

We will describe some methods of improving serial Operator Splitting performance and their effect on accuracy. For example, multinomial direction selection [5], shows some performance gain in our implementation compared to uniform random distribution, but depends strongly on the number of diffusing molecules. This suggests the best approach is to introduce an adaptive algorithm that can switch between solutions depending on simulation conditions. Further we find that, to preserve noise in the system, it is essential to maintain multinomial distribution rather than apply coarser approximations.

Execution of reaction events must be carefully considered in the Operator Splitting method and unique solutions are required to avoid introduction of significant errors. Some previously proposed solutions perform poorly under sensitive conditions. We will discuss the origin of this error and demonstrate how these errors can be reduced by maintaining alignment to the SSA clock. Further, we find convergence of serial Operator Split to the exact spatial SSA with decreasing communication time-step, which however comes at a cost to performance.

Our proposed solution draws on many of these ideas, making several adaptations to simulation conditions, and is tailored for tetrahedral meshes where probability of leap varies locally. As such, our solution has similarities to the MPD-RDME method discussed in [6], but with time-steps adaptive to the upper-limit in the most spatially resolved region, whilst maintaining alignment to the SSA reaction clock. We will demonstrate accuracy by application to our routine reaction-diffusion validation tests [2].
